# Three Characteristics of Cheetah Galloping Improve Running Performance Through Spinal Movement: A Modeling Study

**DOI:** 10.3389/fbioe.2022.825638

**Published:** 2022-04-14

**Authors:** Tomoya Kamimura, Kaho Sato, Shinya Aoi, Yasuo Higurashi, Naomi Wada, Kazuo Tsuchiya, Akihito Sano, Fumitoshi Matsuno

**Affiliations:** ^1^ Department of Electrical and Mechanical Engineering, Nagoya Institute of Technology, Aichi, Japan; ^2^ Department of Aeronautics and Astronautics, Graduate School of Engineering, Kyoto University, Kyoto, Japan; ^3^ Laboratory of System Physiology, Joint Faculty of Veterinary Medicine, Yamaguchi University, Yamaguchi, Japan; ^4^ Department of Mechanical Engineering and Science, Graduate School of Engineering, Kyoto University, Kyoto, Japan

**Keywords:** quadruped, cheetah, galloping, spine bending, simple model

## Abstract

Cheetahs are the fastest land animal. Their galloping shows three characteristics: small vertical movement of their center of mass, small whole-body pitching movement, and large spine bending movement. We hypothesize that these characteristics lead to enhanced gait performance in cheetahs, including higher gait speed. In this study, we used a simple model with a spine joint and torsional spring, which emulate the body flexibility, to verify our hypothesis from a dynamic perspective. Specifically, we numerically searched periodic solutions and evaluated what extent each solution shows the three characteristics. We then evaluated the gait performance and found that the solutions with the characteristics achieve high performances. This result supports our hypothesis. Furthermore, we revealed the mechanism for the high performances through the dynamics of the spine movement. These findings extend the current understanding of the dynamic mechanisms underlying high-speed locomotion in cheetahs.

## 1 Introduction

**FIGURE 1:**
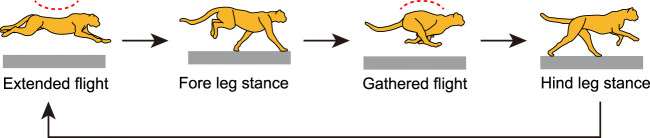
Cheetah galloping involves two types of flight, extended and gathered, which are achieved through spinal movement. Extended and gathered flight occur after liftoff of hindlegs and forelegs, respectively.

Cheetahs are the fastest land animal. They use galloping when moving at their highest speeds, which involves remarkable spine bending movement (Hildebrand, 1959; Bertram and Gutmann, 2009) and stable head height during running. The spine movement allows cheetahs’ gallop to involve two types of flight phase, extended and gathered, as shown in Figure 1 (Hildebrand, 1959, Hildebrand, 1961; English, 1980; Walter and Carrier, 2007; Kamimura et al., 2021). The stable height of the head allows cheetahs to maintain visual contact with their prey, which is achieved not only through a well-designed controller (Grohé et al., 2018), but also through small vertical movements of their center of mass (COM) and small pitching movements of their whole body (Wada, 2011; Ichikawa et al., 2018). The characteristics of cheetah galloping can be summarized as follows: small vertical COM movement, small whole-body pitching movement, and large spine bending movement. We hypothesize that these characteristics provide cheetahs enhanced gait performance, including higher gait speed. However, animal running is a complex phenomenon generated through dynamic interactions between the body’s mechanical systems, nervous system, and the environment; it is difficult to fully understand the mechanisms underlying cheetah galloping only through observational studies.

To overcome the limitations of the observational approach, modeling research approaches have recently attracted attention (Alexander, 1988; Swanstrom et al., 2005; Bertram and Gutmann, 2009; Aoi et al., 2017; Ambe et al., 2018; Fujiki et al., 2018; Aoi et al., 2019; Toeda et al., 2019). Because the legs can be represented by springs, quadruped models with spring legs were developed to investigate the common and unique principles of animal gaits from a dynamic perspective (Full and Koditschek, 1999; Blickhan and Full, 1993; Farley et al., 1993; Tanase et al., 2015; Gan et al., 2016, Gan et al., 2018; Yamada et al., 2022). Recently, such models have been further improved to investigate the dynamic role of spine bending movements in quadruped running (Cao and Poulakakis, 2013; Pouya et al., 2017; Wang et al., 2017; Kamimura et al., 2015, Kamimura et al., 2018; Yesilevskiy et al., 2018; Adachi et al., 2020). Moreover, quadruped robots equipped with a spine bending mechanism have been developed to investigate the effect of spinal movement on running (Pouya et al., 2017; Chen et al., 2019; Fukuhara et al., 2020, Fukuhara et al., 2021). Furthermore, some researchers focused on the impulse from the ground reaction force to reveal the mechanisms underlying various quadruped gaits (Ruina et al., 2005; Usherwood and Davies, 2017; Usherwood, 2020; Polet, 2021; Kamimura et al., 2021). However, because few researchers have focused on the three characteristics of cheetah galloping, the dynamic effects of the characteristics on running and their mechanisms remain largely unclear.

In our previous studies (Kamimura et al., 2015, Kamimura et al., 2018, Kamimura et al., 2021), we used simple models to reveal the mechanisms under which cheetahs utilize their spinal movements to involve the two types of flight phase to achieve high-speed galloping. However, we focused only on spinal movement and did not incorporate the two other characteristics of cheetah galloping: small vertical COM movement and small whole-body pitching movement. In this paper, we extended our previous models to verify our hypothesis that the three characteristics of cheetah galloping enhance gait performance, including gait speed, and clarify their mechanisms. Specifically, we numerically searched for periodic solutions, and evaluated what extent each solution shows the three gait characteristics. In addition, we compared the three characteristics of the solutions with the measured data of actual cheetahs. We then evaluated gait performance to reveal the relationship between the gait characteristics and performance. Furthermore, we compared the solutions with and without the gait characteristics to reveal the mechanisms by which the three characteristics allow cheetahs to achieve high performance when galloping.

## 2 Methods

### 2.1 Model

**FIGURE 2:**
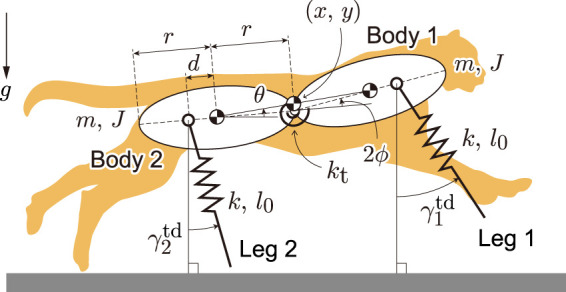
Our model consists of two rigid bodies connected by a joint with torsional spring and two massless spring legs.

To investigate the bounding gait of the model with spine flexibility as in previous studies (Cao and Poulakakis, 2013; Kamimura et al., 2015, Kamimura et al., 2018), we used a two-dimensional model (Figure 2) composed of two rigid bodies (Bodies 1 and 2) and two massless spring legs (Legs 1 and 2). The bodies were connected by a joint, which was modeled to emulate the bending movement of the spine and has a torsional spring with a spring constant of *k*_t_. *x* and *y* represent the horizontal and vertical positions, respectively, of the COM of the whole body. *θ* represents the pitch angle of the line connecting the two COMs of the rigid bodies relative to the horizontal line, that is the pitch angle of the whole body. The spine joint angle is represented by 2*ϕ*. We assumed that the fore and hind parts of the model had the same physical parameters. The mass and moment of inertia around the COM of each body are represented by *m* and *J*, respectively. The length of each body is 2*r*. The distance between the COM and leg joint is *d*, which is positive when the leg joint is outside the COM relative to the spine joint. The torsional spring is at equilibrium position when the fore and hind bodies are in a straight line (*ϕ* = 0). The gravitational acceleration is *g*. The spring constant and nominal length of leg springs are *k* and *l*_0_, respectively. When Leg *i* (*i* = 1, 2) is in the air, its length remains *l*_0_ and its angle keeps the touchdown angle, $\gamma_{i}^{\text{td}}$ .

When the tip of the leg reaches the ground, it is constrained on the ground and behaves as a frictionless pin joint. When the stance leg returns to its nominal length after compression, the tip leaves the ground, and the leg angle immediately returns to the touchdown angle $\gamma_{i}^{\text{td}}$ . The model conserves energy because the touchdown and liftoff occur at the nominal length and the model has no dissipative structure, such as friction or a damper.

The equations of motion of the model are given by

$$M\left(q\right)\ddot{q}+h\left(q,\dot{q}\right)+v\left(q\right)=0$$ (1)

where *q* = [*x y θ ϕ*]^*⊤*^,

$$\begin{array}{ll} M\left(q\right) & =\left[\begin{array}{cccc} 2m & 0 & 0 & 0\\ 0 & 2m & 0 & 0\\ 0 & 0 & 2J+{2mr}^{2}\cos^{2}\phi & 0\\ 0 & 0 & 0 & {2J+2mr}^{2}\sin^{2}\phi \end{array}\right],\\ h\left(q,\dot{q}\right) & =\left[\begin{array}{c} 0\\ 0\\ -4mr^{2}\dot{\theta }\dot{\phi }\cos\phi \sin\phi \\ 2mr^{2}\left({\dot{\theta }}^{2}+{\dot{\phi }}^{2}\right) \end{array}\right],\\ v\left(q\right) & =\left[\begin{array}{c} 0\\ 2mg\\ 0\\ 4k_{\text{t}}\phi \end{array}\right]+k\left(l_{1}-l_{0}\right)\left[\begin{array}{c} \partial l_{1}/\partial x\\ \partial l_{1}/\partial y\\ \partial l_{1}/\partial \theta \\ \;\partial l_{1}/\partial \phi \end{array}\right]+k\left(l_{2}-l_{0}\right)\left[\begin{array}{c} \partial l_{2}/\partial x\\ \partial l_{2}/\partial y\\ \partial l_{2}/\partial \theta \\ \;\partial l_{2}/\partial \phi \end{array}\right], \end{array}$$

$$l_{i}=\{\begin{array}{c} l_{0},\;when\;Leg\;i\;is\;in\;the\;air\;\\ {}[{\left\{x+{\left(-1\right)}^{i}\left(r+d\right)\cos\theta \cos\phi -x_{i}^{\text{toe}}\right\}}^{2}\\ +{\left\{y+{\left(-1\right)}^{i}\left(r+d\right)\sin\theta \cos\phi \right\}}^{2}{]}^{\frac{1}{2}},\\ \;when\;Leg\;i\;is\;in\;the\;stance\;phase\; \end{array}i=1,2$$

and $x_{i}^{\text{toe}}$ is the contact position of Leg *i*.

From the measured cheetah data (Kamimura et al., 2021), we determined the physical parameters as follows: *m* = 19 kg, *J* = 0.53 kgm^2^, *r* = 0.29 m, *l*_0_ = 0.69 m, and *d* = 0.06 m. We used *k*_t_ = 100 Nm/rad and *k* = 15000 N/m to reproduce a similar locomotor behavior to that of cheetahs from the perspective of body bending and gait cycle.

### 2.2 Search for Periodic Solutions

**FIGURE 3:**
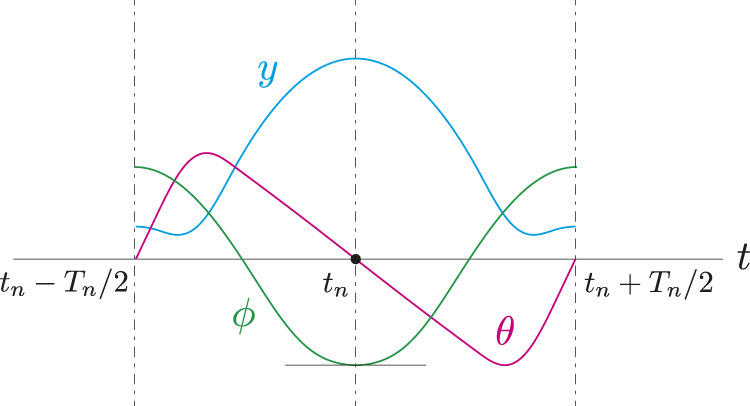
Additional constraints *θ*(*t*_*n*_) = 0 and $\dot{\phi }\left(t_{n}\right)=0$ to achieve symmetrical periodic solutions. *y*, *θ*, and *ϕ* are COM height, whole-body pitch angle, and relative angle between Bodies 1 and 2, respectively.

To find periodic solutions of the model, we defined the Poincaré section at the apex height of the COM of the whole body $\left(\dot{y}=0\right)$ . We set *t* = *t*_*n*_ at the *n*th apex height. We assumed that both legs have to experience the stance phase once before the next intersection with the Poincaré section. We neglected the horizontal position to determine periodic solutions because it monotonically increases during locomotion and is not periodic. We assumed the following two constraints to movement based on Raibert (1986):

$$\theta \left(t_{n}\right)=0,$$ (2a)

$$\dot{\phi }\left(t_{n}\right)=0,$$ (2b)

so that the whole-body posture is symmetrical about a vertical axis through the spine joint and that the body spring is fully bent at the apex height, as shown in Figure 3. This allows symmetrical periodic solutions to be achieved that satisfy

$$y\left(t_{n}+\tau \right)=y\left(t_{n}-\tau \right),$$ (3a)

$$\phi \left(t_{n}+\tau \right)=\phi \left(t_{n}-\tau \right),$$ (3b)

$$\theta \left(t_{n}+\tau \right)=-\theta \left(t_{n}-\tau \right),$$ (3c)

where *t*_*n*_ − *T*_*n*_/2 ≤ *τ* ≤ *t*_*n*_ + *T*_*n*_/2 and *T*_*n*_ = *t*_*n*+1_ − *t*_*n*_.

We used the Poincaré map *P* denoted by

$$z_{n+1}=P\left(z_{n},u_{n}\right),$$ (4)

where $z_{n}=\left[y\left(t_{n}\right)\;\phi \left(t_{n}\right)\;\dot{\theta }\left(t_{n}\right)\right]$ was the state variable at the *n*th intersection with the Poincaré section and $u_{n}=\left[\gamma_{1n}^{\text{td}}\;\gamma_{2n}^{\text{td}}\right]$ was the parameter set. To find the solutions, we fixed the total energy *E*, and $\dot{x}\left(t_{n}\right)$ was determined using other variables. For a periodic solution, z* = *P*(z*, u*) is satisfied, where z* is a fixed point on the Poincaré section. We numerically searched for fixed points for periodic solutions using the Newton–Raphson method.

### 2.3 Classification of Solutions

**FIGURE 4:**
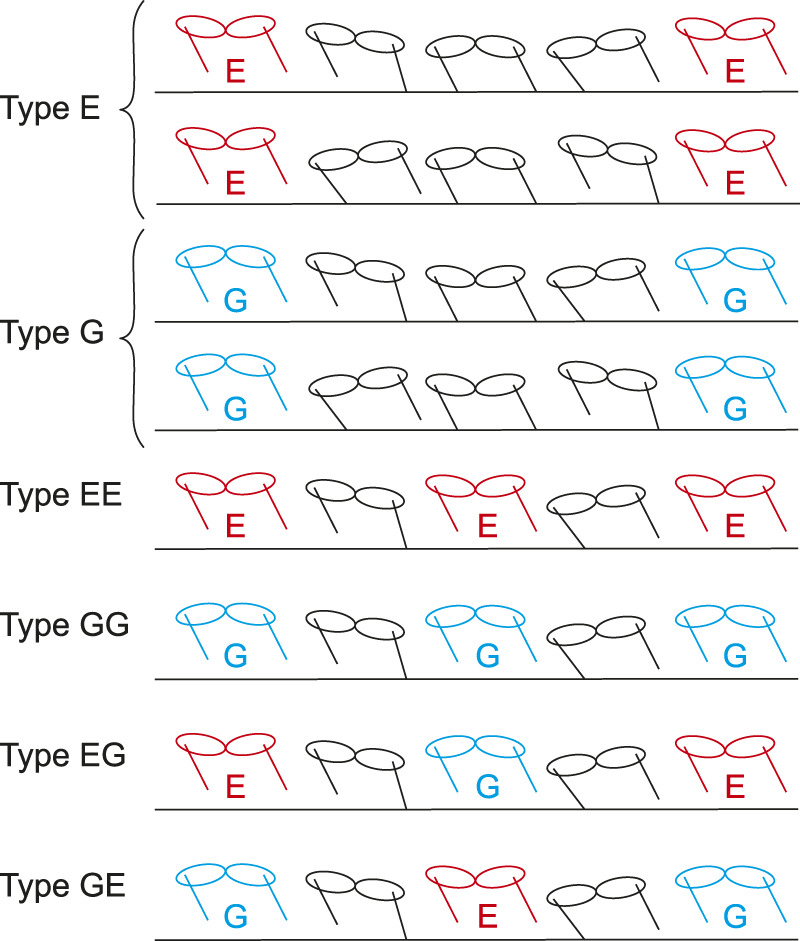
Six types of periodic solution.

In the obtained periodic solutions, some solutions only had one flight phase and one double stance phase. Other solutions had two flight phases but no double stance phase. In addition, the flight phases are classified into two types based on the spine joint movement: extended and gathered. In extended flight, the spine joint is extended (*ϕ* > 0) at the mid-flight phase. In gathered flight, the spine joint is flexed (*ϕ* < 0) at the mid-flight phase. As a result, periodic solutions are classified into six types as shown in Figure 4:

1 Type E: Single extended flight with double stance
2 Type G: Single gathered flight with double stance
3 Type EE: Two extended flights without double stance
4 Type GG: Two gathered flights without double stance
5 Type EG: Two different flights (first: extended, second: gathered) without double stance
6 Type GE: Two different flights (first: gathered, second: extended) without double stance

where we assumed that the foreleg (Leg 1) is the first to touch down in solutions with two flights (Types EE, GG, EG, and GE).

### 2.4 Gait Characteristics of Cheetahs

Cheetah galloping shows three characteristics: small vertical COM movement, small whole-body pitching movement, and large spine bending movement. To evaluate to what extent the obtained periodic solutions showed these characteristics, we investigated the fluctuations of the COM height *δ*_*y*_, whole-body pitch angle *δ*_*θ*_, and spine joint angle *δ*_*ϕ*_, by examining the difference between the maximum and minimum values of *y*(*t*), *θ*(*t*), and *ϕ*(*t*), respectively, in one gait cycle as follows:

$$\delta_{y}=\underset{t_{n}\leq t\leq t_{n}+T_{n}}{\max}y\left(t\right)-\underset{t_{n}\leq t\leq t_{n}+T_{n}}{\min}y\left(t\right),$$ (5)

$$\delta_{\theta }=\underset{t_{n}\leq t\leq t_{n}+T_{n}}{\max}\theta \left(t\right)-\underset{t_{n}\leq t\leq t_{n}+T_{n}}{\min}\theta \left(t\right),$$ (6)

$$\delta_{\phi }=\underset{t_{n}\leq t\leq t_{n}+T_{n}}{\max}\phi \left(t\right)-\underset{t_{n}\leq t\leq t_{n}+T_{n}}{\min}\phi \left(t\right).$$ (7)

We compared these three characteristics with measured cheetah data in Kamimura et al. (2021), where four adult male cheetahs (40–50 kg) ran at a speed of 15–18 m/s. We analyzed eight strides (five from one cheetah and three from the others) and determined *δ*_*y*_ by the fluctuation of the height of the middle point between the shoulder joint and the greater trochanter of the femur, *δ*_*θ*_ by the fluctuation of the pitch angle of the line connecting the roots of the head and the tail, and *δ*_*ϕ*_ by the fluctuation of the relative angle between the lines connecting the root of neck and 12th thoracic vertebra (T12), and T12 and root of the tail.

### 2.5 Performance Criteria

To evaluate the gait performance of the obtained solutions, we used the following three criteria: average horizontal velocity, impulse from the ground reaction force, and stability. The average horizontal velocity for one gait cycle of the periodic solution was calculated as

$$\bar{v}=\frac{1}{T_{n}}\int_{t_{n}}^{t_{n}+T_{n}}\dot{x}\left(t\right)dt=\frac{x\left(t_{n}+T_{n}\right)-x\left(t_{n}\right)}{T_{n}}.$$ (8)

We evaluated the following aspects of the impulse from the ground reaction force: net, positive horizontal, negative horizontal, and vertical impulse. The net impulse for one gait cycle from the foreleg was identical to that from the hind-leg due to the symmetry of the motion and was obtained by

$$p=-\int_{t_{n}}^{t_{n}+T_{n}}k\left(l_{0}-l_{i}\left(t\right)\right)dt\;i=1,2$$ (9)

In the stance phase of Leg *i* (*i* = 1, 2), when *γ*_*i*_ > 0, the horizontal ground reaction force reduces the horizontal velocity of the COM. In contrast, it increases when *γ*_*i*_ < 0. Because $\gamma_{i}^{\text{td}}>0$ is satisfied for all obtained periodic solutions, we assumed that *γ*_*i*_ monotonically decreases and liftoff occurs with *γ*_*i*_ < 0. The negative horizontal impulse $p_{i}^{x-}$ and positive horizontal impulse $p_{i}^{x-}$ were then obtained by

$$p_{i}^{x-}=-\int_{t_{n}}^{t_{n}+T_{ni}}k\left(l_{0}-l_{i}\left(t\right)\right)\sin\gamma_{i}\left(t\right)dt,\;i=1,2$$ (10)

$$p_{i}^{x+}=-\int_{t_{n}+T_{ni}}^{t_{n}+T_{n}}k\left(l_{0}-l_{i}\left(t\right)\right)\sin\gamma_{i}\left(t\right)dt,\;i=1,2$$ (11)

where *t*_*n*_ + *T*_*ni*_ is the moment when *γ*_*i*_ = 0 is achieved. Note that the amount of acceleration by the foreleg and deceleration by the hind-leg are identical, and the acceleration by the hind-leg and deceleration by the foreleg are identical. In other words, $p_{i}^{x-}=-p_{j}^{x+}$ is satisfied for (*i*, *j*) = (1, 2) and (2, 1). The vertical impulse from the foreleg is identical to that from the hind-leg and was obtained by

$$p^{y}=\int_{t_{n}}^{t_{n}+T_{n}}k\left(l_{0}-l_{i}\left(t\right)\right)\cos\gamma_{i}\left(t\right)dt\;i=1,2$$ (12)

The stability was determined by the eigenvalues of the linearized Poincaré map around the fixed point on the Poincaré section. Because our model was energy conservative, the solution was asymptotically stable when all of the eigenvalues, except for one eigenvalue of 1, were inside the unit cycle in the complex plane (these magnitudes are less than 1).

## 3 Results

### 3.1 Periodic Solutions

**FIGURE 5:**
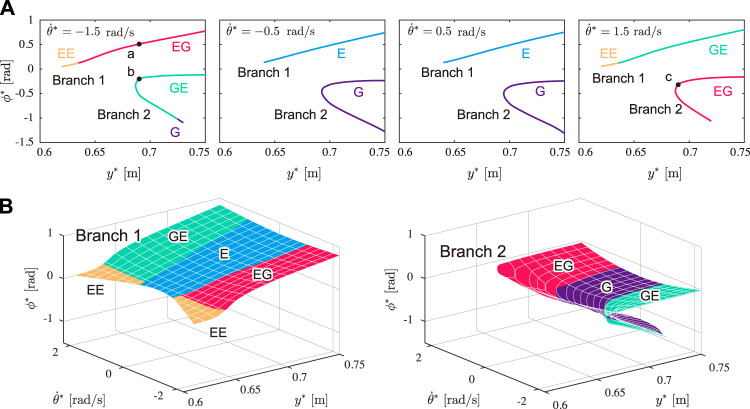
Obtained solutions (Type E: blue, Type G: purple, Type EE: yellow, Type EG: red, and Type GE: green). **(A)** Solutions for ${\dot{\theta }}^{*}=-1.5,-0.5,0.5$ , and 1.5 rad/s. Black dots (a, b, and c) indicate the solutions used in Figure 6. **(B)** Solutions plotted in y*- ${\dot{\theta }}^{*}$ -ϕ* space. Left and right figures show Branches 1 and 2, respectively.

We numerically searched for periodic solutions using *E*_0_ = 4500 J to achieve a gait speed similar to that of cheetah galloping. Figure 5A shows the obtained solutions for ${\dot{\theta }}^{*}=-1.5,-0.5,0.5$ , and 1.5 rad/s. Although five types of solutions (Types E, G, EE, EG, and GE) were found, Type GG was not. Regardless of the value of ${\dot{\theta }}^{*}$ , the solutions could be divided into two parts: Branch 1 (upper) and Branch 2 (lower). While Branch 1 was obtained over a wide range of y*, Branch 2 was obtained for a smaller range and folded. In other words, Branch 2 had two solutions for each y* in the specific range. Figure 5B shows the obtained solutions in the y*- ${\dot{\theta }}^{*}$ -ϕ* space, where the left and right figures show Branches 1 and 2, respectively. When ${\dot{\theta }}^{*}$ was small, the solutions involved only one flight phase (Types E and G). When ${\dot{\theta }}^{*}$ was large, the solutions had two different flight phases (Types EG, GE, and EE). Type EE existed only in Branch 1, where $\left|{\dot{\theta }}^{*}\right|$ is large and y* is small.

**FIGURE 6:**
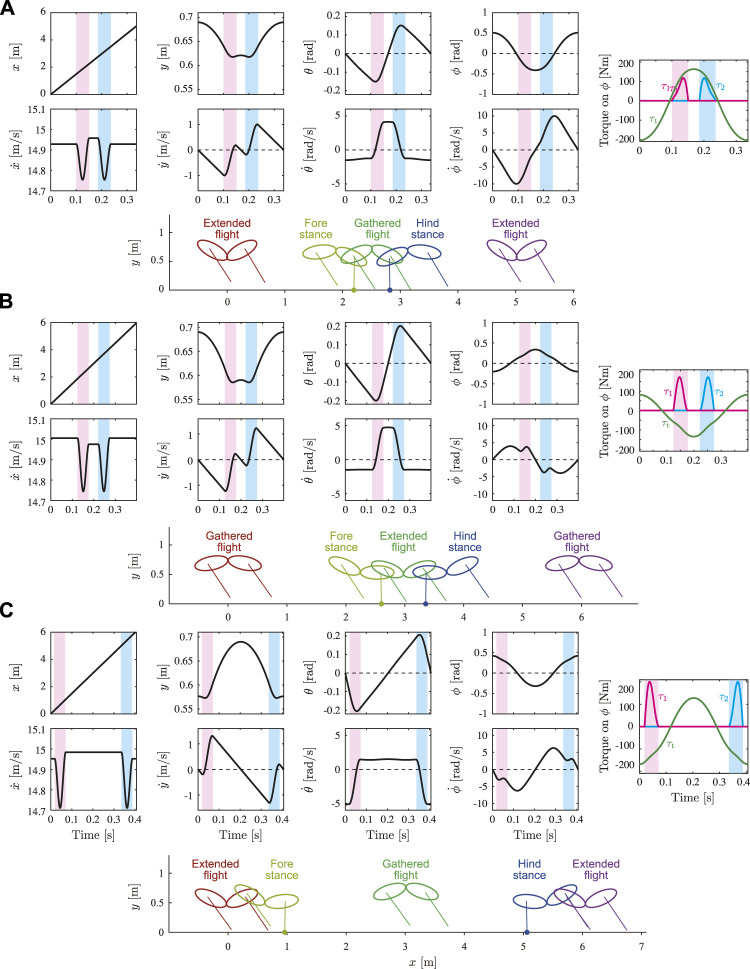
Time profiles and snapshots of typical solutions for y* = 0.69 m. Snapshots illustrate state at mid-stance or mid-flight in each phase (see Supplementary Movie). **(A)** Solution in Branch 1 (Type EG, a in Figure 5) and **(B)** solution in Branch 2 (Type GE, b in Figure 5) for ${\dot{\theta }}^{*}=-1.5$ rad/s. **(C)** Solution in Branch 2 (Type EG, c in Figure 5) for ${\dot{\theta }}^{*}=1.5$ rad/s. Red and blue shaded areas indicate stance phase of foreleg and hind-leg, respectively.

Figure 6 shows the time profiles and snapshots of typical solutions for y* = 0.69 m. Figures 6A,B show the solution in Branch 1 (Type EG, a in Figure 5A) and one of the solutions in Branch 2 with smaller |ϕ*| (Type GE, b in Figure 5A), respectively, for ${\dot{\theta }}^{*}=-1.5$ rad/s. Figure 6C shows one of the solutions in Branch 2 with smaller |ϕ*| (Type EG, c in Figure 5A) for ${\dot{\theta }}^{*}=1.5$ rad/s.

When we compare the time profiles of the state variables between Figures 6A,B, there is no qualitative difference in *x*, *y*, *θ*, $\dot{y}$ , and $\dot{\theta }$ . In contrast, the signs of *ϕ* and $\dot{\phi }$ are different, which results in different solution types (Types EG and GE). In addition, the time profiles of $\dot{x}$ are partially different. Specifically, while $\dot{x}$ during the flight phase before touchdown of the foreleg is larger than that after the touchdown of the hind-leg in Figure 6A, it is smaller in Figure 6B. Furthermore, while $\dot{\phi }$ has only one peak in Figure 6A, it has three peaks in Figure 6B. When we compare the time profiles of the state variables between Figures 6A,C, the durations of two flights are different despite the same solution type (Type EG). Specifically, while the duration of the gathered flight (second flight) is shorter than that of extended flight (first flight) in Figure 6A, it is longer in Figure 6C. Furthermore, the time profiles of *ϕ* and $\dot{\phi }$ are also different. Specifically, in the stance phases in Figure 6A, *ϕ* < 0, in Figure 6C, *ϕ* > 0. In addition, while $\dot{\phi }$ has only one peak in Figure 6A, it has three peaks in Figure 6C.

To understand the mechanism underlying the differences between Branches 1 and 2, we compared the torque on the spine joint *ϕ* in the stance phases. While the joint torque *τ*_t_ on *ϕ* from the body spring (first term in the fourth row of *v*(*q*) in Eq. 1) is positive to extend the spine joint in the stance phases in Figure 6A, it is negative to bend the spine joint in Figures 6B,C. The moments *τ*_1_ and *τ*_2_ on *ϕ* from the ground reaction forces of Legs 1 and 2, respectively (second and third terms, respectively, in the fourth row of *v*(*q*) in Eq. 1), are both positive to extend the spine joint.

### 3.2 Gait Characteristics of Solutions

**FIGURE 7:**
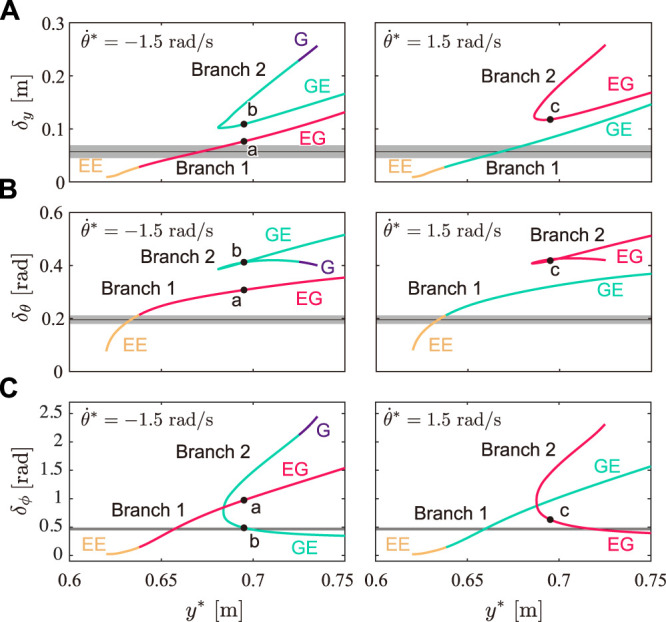
Gait characteristics of solutions. Fluctuations of **(A)** COM height *δ*_*y*_, **(B)** whole-body pitch angle *δ*_*θ*_, and **(C)** spine joint angle *δ*_*ϕ*_ for y* for solutions with ${\dot{\theta }}^{*}=\pm 1.5$ rad/s. Black dots (a, b, and c) indicate the solutions in Figure 5A. Black lines and grey areas show average values and standard errors, respectively, of eight strides of measured cheetah data.

We quantitatively evaluated what extent each obtained solution shows the characteristics of cheetah galloping. Figures 7A–C show the fluctuations in the COM height *δ*_*y*_, whole-body pitch angle *δ*_*θ*_, and spine joint angle *δ*_*ϕ*_, respectively, for y* of the obtained solutions with ${\dot{\theta }}^{*}=\pm 1.5$ rad/s. *δ*_*y*_ and *δ*_*θ*_ of Branch 1 were smaller than those of Branch 2 for each y*, regardless of ${\dot{\theta }}^{*}$ . In contrast, Branch 2 involved a larger *δ*_*ϕ*_ and smaller *δ*_*ϕ*_ than that of Branch 1 for each y*. When we compare the solutions indicated by a (Branch 1) and b (Branch 2) in Figure 5A, solution a has a larger *δ*_*ϕ*_.

From the measured cheetah data, we obtained *δ*_*y*_ = 0.057 ± 0.012 (S.E.) m, *δ*_*θ*_ = 0.20 ± 0.016 rad, and *δ*_*ϕ*_ = 0.47 ± 0.029 rad, as shown in Figure 7, where black lines and grey areas show the average values and standard errors, respectively, of eight strides of the measured cheetah data. The solutions of Branch 1 showed closer values for *δ*_*y*_, *δ*_*θ*_, and *δ*_*ϕ*_ to those of the measured data than the solutions of Branch 2.

### 3.3 Gait Performance of Solutions

**FIGURE 8:**
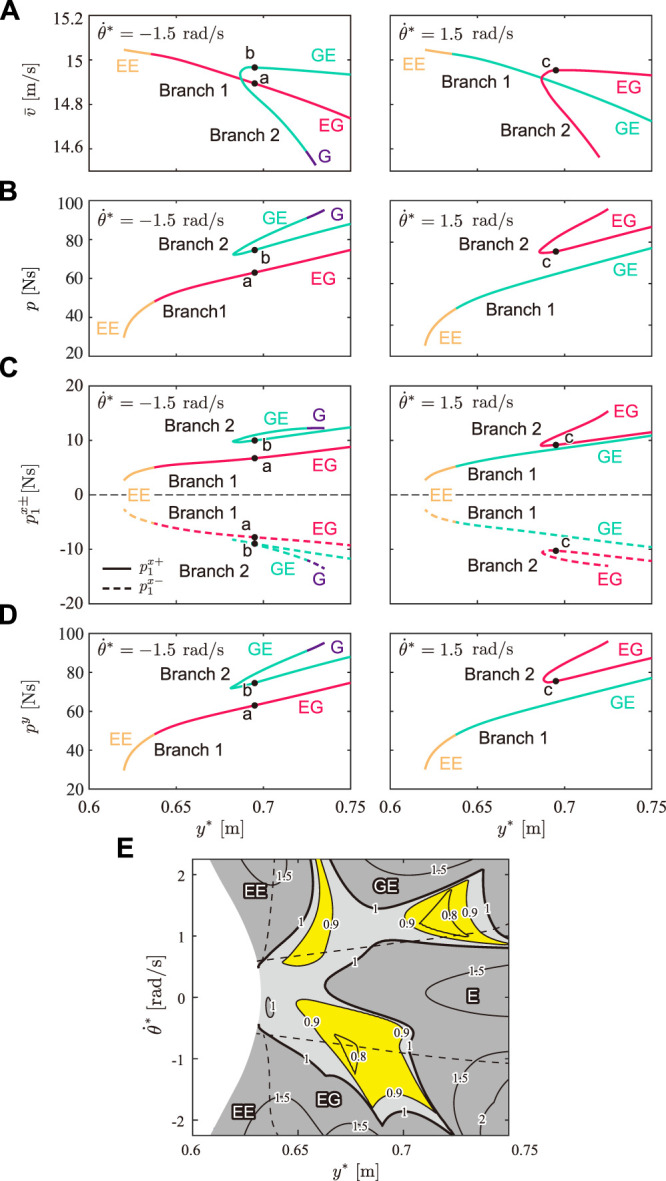
Gait performance of solutions. **(A)** Averaged horizontal velocity $\bar{v}$ , **(B)** net impulse *p*, **(C)** horizontal impulses $p_{1}^{x+}$ and $p_{1}^{x-}$ , and **(D)** vertical impulse *p* ^*y*^ for y* of solutions with ${\dot{\theta }}^{*}=\pm 1.5$ rad/s. Black dots (a, b, and c) indicate the solutions in Figure 5A. **(E)** Contour of maximum eigenvalue of solutions in Branch 1. Dotted lines indicate boundaries of solution types. No solution was found in white area. When maximum eigenvalue is less than 1, solution is stable (light grey and yellow areas). Dark grey areas indicate unstable solutions, which have maximum eigenvalues greater than 1. Yellow areas indicate stable solutions with maximum eigenvalue less than 0.9.

We evaluated the gait performance of the obtained solutions by focusing on the difference between the two branches. Figure 8A shows the average horizontal velocity $\bar{v}$ of the solutions found for ${\dot{\theta }}^{*}=\pm 1.5$ rad/s. It shows 14.5–15 m/s, which is consistent with the velocity range of cheetah galloping (Hudson et al., 2012). It monotonically increases as y* decreases in Branch 1. Branch 2 involves a larger $\bar{v}$ and smaller $\bar{v}$ than those of Branch 1 for each y*. However, Branch 1 has a larger range of y* than Branch 2 and the maximum $\bar{v}$ of Branch 1 is slightly larger than that of Branch 2.

Figures 8B–D show the net impulse *p*, horizontal impulses $p_{1}^{x+}$ and $p_{1}^{x-}$ , and vertical impulse *p* ^*y*^, respectively, for ${\dot{\theta }}^{*}=\pm 1.5$ rad/s. The horizontal impulses $p_{2}^{x+}$ and $p_{2}^{x-}$ are not shown because $p_{2}^{x+}=-p_{1}^{x-}$ and $p_{2}^{x-}=-p_{1}^{x+}$ are satisfied. For all impulses, Branch 1 involves smaller absolute values than those of Branch 2.

Branch 2 has no stable solution and stable solutions were found only in Branch 1. Figure 8E shows the contour of the maximum eigenvalue of the linearized Poincaré map around the fixed point on the Poincaré section of Branch 1 projected onto the y*- ${\dot{\theta }}^{*}$ plane. Only Types E, EG, and GE have stable solutions. No solutions were found in the white area. In addition, ${\dot{\theta }}^{*}<0$ has a larger area where the maximum eigenvalue is smaller than 0.9 than ${\dot{\theta }}^{*}>0$ .

## 4 Discussion

Cheetahs are the fastest land animals. Their galloping shows three characteristics: small fluctuations in the COM height and whole-body pitch angle, and notable spinal bending. In this study, to clarify the dynamic mechanisms underlying these characteristics, we constructed a simple model and numerically obtained periodic solutions. The obtained solutions were classified into six types with respect to their flight phase and the spine joint movement. Solutions of Types E and G involve single flight with double stance, whose flight is extended and gathered, respectively. Solutions of Types EE and GG involve two flights without double stance, whose flights are both extended and both gathered, respectively. Solutions of Types EG and GE involve two flights without double stance, whose flights are extended and gathered like cheetahs. While the first and second flights are extended and gathered, respectively, for solutions of Type EG, they are gathered and extended for solutions of Type GE.

### 4.1 Comparison of Gait Characteristics and Performance Between Branches 1 and 2

The obtained periodic solutions were classified into two branches: Branch 1 and Branch 2 (Branch 2 has two solutions for each y*, as shown in Figure 5A). We compared the three characteristics in cheetah galloping between the solutions of Branches 1 and 2. The solutions of Branch 1 involved smaller fluctuations of the COM height *δ*_*y*_ and whole-body pitch angle *δ*_*θ*_ than those of both solutions of Branch 2 for each y*, as shown in Figures 7A,B, respectively. Moreover, the solutions of Branch 1 were obtained over a wider range of apex COM height y* than the solutions of Branch 2, as shown in Figure 5A. Therefore, the solutions of Branch 1 showed smaller vertical movement of the COM and whole-body pitching movement than the solutions of Branch 2, which is consistent with cheetah galloping. Furthermore, the two solutions of Branch 2 for each y* involve larger and smaller fluctuations of the spine joint angle *δ*_*ϕ*_ than that of the solution of Branch 1 (Figure 7C). However, the solutions of Branch 2 with larger *δ*_*ϕ*_ involve larger *δ*_*y*_ (Figure 7A), smaller average horizontal velocity $\bar{v}$ (Figure 8A), and larger net impulse *p* (Figure 8B) than those of the other solutions, which indicates that although the solutions involve notable spinal bending, they do not show the characteristics of cheetah galloping (this mechanism is discussed in the next section). The solutions of Branch 1 have sufficiently larger *δ*_*ϕ*_ than the solutions of Branch 2 with smaller *δ*_*ϕ*_, which implies that the solutions of Branch 1 also show the third characteristic (large spine bending movement) well. Therefore, the solutions of Branch 1 clearly show the characteristics of cheetah galloping than the solutions of Branch 2.

We next compared the three gait performances between the solutions of Branches 1 and 2. Although the solution of Type EE in Branch 1 involves the maximum average horizontal velocity $\bar{v}$ (Figure 8A), the solutions of Branch 1 have larger $\bar{v}$ than the solutions of Branch 2, even when we focus on the solutions of Types EG and GE, which involve two types of flight phase in one gait cycle, similar to cheetah galloping. Furthermore, the solutions of Branch 1 have smaller net impulse *p* from the ground reaction force than the solutions of Branch 2 (Figure 8B). Moreover, while Branch 1 involves stable solutions (Figure 8E), Branch 2 does not. These results show that the solutions in Branch 1, which show the characteristics of cheetah galloping well, lead to better gait performance than those of Branch 2.

### 4.2 Mechanisms for Different Characteristics and Performance Between Branches 1 and 2

**FIGURE 9:**
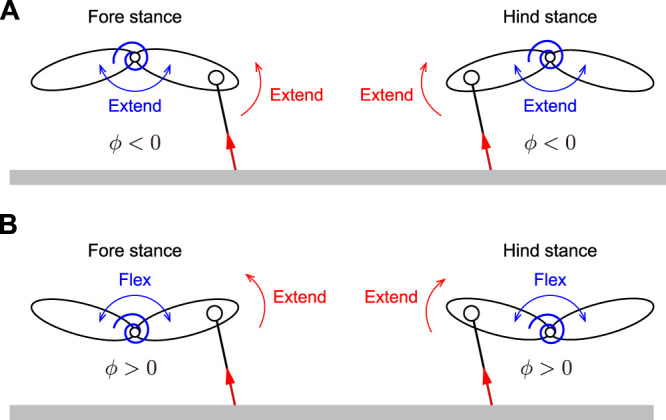
Contribution of body spring and ground reaction force to spine joint dynamics. **(A)** Both body spring and ground reaction force extend spine joint in solutions of Branch 1. **(B)** While body spring flexes spine joint, ground reaction force extends spine joint in solutions of Branch 2.

An important difference between the solutions of Branches 1 and 2 appears in the directions of the joint torque *τ*_t_ on the spine joint *ϕ* by the body spring, and the moments *τ*_1_ and *τ*_2_ on *ϕ* from the ground reaction forces of Legs 1 and 2, respectively. *τ*_1_ and *τ*_2_ extend the spine joint regardless of Branch (Figures 6, 9). In contrast, the direction of *τ*_t_ depends on the Branch. Specifically, while *τ*_t_ extends the spine joint in the stance phase of both the foreleg and hind-leg in the solutions of Branch 1 because *ϕ* < 0 (Figure 6A), it bends in the solutions of Branch 2 because *ϕ* > 0 (Figures 6B,C). Therefore, while the directions of *τ*_t_ and *τ*_*i*_ (*i* = 1, 2) are the same in the solutions of Branch 1, they are opposite in the solutions of Branch 2. This difference explains that the ground reaction forces enhance spinal movement in the solutions of Branch 1 whereas they prevent in the solutions of Branch 2. In addition, the opposite direction induced three peaks in $\dot{\phi }$ in the solutions of Branch 2 (Figures 6B,C).

Because of the different directions of the torque on the spine joint described above, while the body spring pushes the legs down in the solutions of Branch 2, it pulls the legs up in the solutions of Branch 1, which made the net impulse *p* of the solutions of Branch 2 larger than that of the solutions of Branch 1 (Figure 8B). Furthermore, the horizontal impulses $\left|p_{1}^{x-}\right|$ and $p_{1}^{x+}$ of the solutions of Branch 2 are also larger than those of the solutions of Branch 1 (Figure 8C). The COM is decelerated by $p_{i}^{x-}$ in the first half of the stance phase of Leg *i* (*i* = 1, 2) (*γ*_*i*_ > 0) and accelerated by $p_{i}^{x+}$ in the second half of the stance phase (*γ*_*i*_ < 0). Because the solutions of Branch 2 have larger values of $\left|p_{i}^{x-}\right|$ and $p_{i}^{x+}$ than the solutions of Branch 1, the COM of the solutions of Branch 2 undergoes greater deceleration and acceleration than that of the solutions of Branch 1. Therefore, even if the solutions of Branches 1 and 2 have the same horizontal velocity in the flight phases, the solutions of Branch 1 have a higher average horizontal velocity $\bar{v}$ than the solutions of Branch 2. Furthermore, the solutions of Branch 1 had a wider range of y* than the solutions of Branch 2 (Figure 5A). When the total energy is identical, the decrease in potential energy leads to an increase in kinetic energy. When ${\dot{\theta }}^{*}$ is also identical, which results in the same rotational kinetic energy in the solutions of Branches 1 and 2, the solutions of Branch 1 can have more translational kinetic energy than the solutions of Branch 2, which results in higher $\bar{v}$ . These explain why the solutions of Branch 1 involve higher average horizontal velocities than the solutions of Branch 2, although the solutions of Branch 1 involve smaller impulses from the ground reaction forces than the solutions of Branch 2. It has been also suggested that spinal movement enhances gait speed because it allows cheetahs to swing their limbs further and increase their stride length (Hildebrand, 1959, Hildebrand, 1961; English, 1980; Schilling and Hackert, 2006; Walter and Carrier, 2007).

The mechanism under which the solutions of Branch 1 showed the characteristics of cheetah galloping can be explained by the difference in the impulses between the solutions of Branches 1 and 2. When the model receives a large vertical impulse *p* ^*y*^, the model lifts off the ground with a large momentum in the *y* direction, which results in a large change in the COM height *δ*_*y*_. Because the solutions of Branch 1 had a smaller vertical impulse *p* ^*y*^ than the solutions of Branch 2 (Figure 8D), the solutions of Branch 1 involved a smaller *δ*_*y*_ than that of the solutions of Branch 2 (Figure 7A). Furthermore, in the solutions without a double stance phase (Types EE, EG, and GE), when the model receives a small impulse, the model lifts off the ground with a small angular momentum in the *θ* direction, which results in a small fluctuation of the whole-body pitch angle *δ*_*θ*_. Therefore, because the solutions of Branch 1 had smaller impulses than the solutions of Branch 2, the solutions of Branch 1 involved smaller *δ*_*θ*_ than the solutions of Branch 2 (Figure 7B). When we focus on solutions with a double stance phase (Type G), *δ*_*θ*_ is smaller than that of the solution of Type GE of Branch 2 (Figure 7B), even when the solutions of Type G have larger impulse *p* than the solutions of Type GE of Branch 2 (Figure 8B). This is because the direction of moments from the ground reaction forces on the forelegs and hindlegs are in opposite directions and these effects are canceled in the double stance phase. However, the *δ*_*θ*_ of the solutions of Branch 2 is still larger than that of the solutions of Branch 1 (Figure 7B) because *p* of the solutions of Branch 1 is smaller than that of the solutions of Branch 2 (Figure 8B).

Moreover, since both the body spring and ground reaction force generate moments to extend the spine joint in the solutions of Branch 1 (Figure 9A), spinal movement is enhanced and results in larger fluctuations of the spine joint angle *δ*_*ϕ*_ than those in the solutions of Branch 2 with smaller *δ*_*ϕ*_ (Figure 7C), where the moment from the ground reaction force prevents spinal movement (Figure 9B). Although spinal movement in the solutions of Branch 2 with a larger *δ*_*ϕ*_ is also prevented, they have larger *δ*_*ϕ*_ than the solutions of Branch 1. This is because while the spine bending in the solutions of Branch 2 with small *δ*_*ϕ*_ mainly appears in the stance phase, which is prevented by the ground reaction forces, the spine bending in the solutions of Branch 2 with large *δ*_*ϕ*_ appears in the flight phase, which is not prevented by ground reaction forces. However, the impulse of the solutions of Branch 2 with larger *δ*_*ϕ*_ is larger than that of the solutions of Branch 1 because the directions of torque from the body spring and ground reaction force are opposite, similar to the solutions of Branch 2 with smaller *δ*_*ϕ*_.

From the discussion above, we revealed the mechanism of why the solutions of Branch 1, which showed the characteristics of cheetah galloping well, achieved better gait performances than those of the solutions of Branch 2. Specifically, the ground reaction forces of the solutions of Branch 1 were reduced through the spine bending movement, which results in high-speed and stable locomotion, even they involved small impulses on the legs.

### 4.3 Why Cheetahs Use Type EG

The cheetah gallop is also characterized by involving two types of flights: extended and gathered (Hildebrand, 1989). In their galloping, the forelegs touch the ground after extended flight, and the gathered flight follows the liftoff of the forelegs. The hindlegs touch the ground after gathered flight, and extended flight follows the liftoff of the hindlegs (Figure 1). Therefore, the solutions of Type EG correspond to the cheetah galloping, as shown in Figure 4.

We obtained not only the solutions of Type EG but also the solutions of Type GE for both Branches 1 and 2, which also have two different flights, but in which the foreleg touches the ground after the gathered flight and the foreleg touches the ground after the extended flight (Figure 4). Although the solutions of Types E, G, EE were also found, no solution of only Type GG was found. This is consistent with Kamimura et al. (2021), where a simple model did not have any solutions of Type GG when *d* > 0, where *d* is the distance between the COM of the body and leg joint (Figure 2). When we compare the solutions of Types EG and GE of Branch 1, although there is no significant difference in the maximum values of the horizontal velocity $\bar{v}$ (Figure 8A), the net impulse *p* of the solutions of Type EG was slightly smaller than that of the solutions of Type GE (Figure 8B). When we focus on the gait stability, although the solutions of Type GE have a larger stable area for y* and ${\dot{\theta }}^{*}$ than that of the solutions of Type EG, the solutions of Type EG have a larger area, where the maximum eigenvalue is less than 0.9, and are more stable than the solutions of Type GE (Figure 8E). These results suggest a reason why cheetahs prefer a gait corresponding to the solutions of Type EG over a gait corresponding to the solutions of Type GE.

### 4.4 Limitations and Future Works

As shown in the solutions of Branch 1 (Figure 6), while $\dot{x}$ is larger after the liftoff of the foreleg than that before the touchdown of the foreleg, it is smaller after the liftoff of the hind-leg than that before the touchdown of the hind-leg. This indicates that the forelegs and hindlegs contribute to acceleration and deceleration, respectively. In contrast, it is reported that the forelegs and hindlegs contribute to deceleration and acceleration, respectively, in actual cheetah galloping (Bertram and Gutmann, 2009). Since $\dot{x}$ is smaller after the liftoff of the foreleg than that before the touchdown of the foreleg and it is larger after the liftoff of the hind-leg than that before the touchdown of the hind-leg, the solutions of Branch 2 are rather consistent with cheetah galloping from the viewpoint of the roles of acceleration and deceleration of the forelegs and hindlegs. This discrepancy is possibly because our model neglected the mass of the legs and the asymmetry of the fore and hind parts of the body. We would like to consider these effects in future research.

Furthermore, the solutions of Branch 1, which show three characteristics of cheetah galloping, have the same magnitude of flexion and extension of the spine (Figure 6). In contrast, the spinal hyperextension caused by the hind leg extension is inhibited by the epaxial muscles, which prevents the loss of propulsive forces by the hind legs in cheetahs (English, 1980; Wada et al., 2010). In addition, the hyperextension of the spinal column is prevented by the spinous processes. This discrepancy is mainly because our model has the same stiffness for the flexion and extension in the body spring, which does not prevent the hyperextension of the spine unlike the epaxial muscles and spinal processes of actual cheetahs. Moreover, although we assumed that the body spring is at the equilibrium position when the fore and hind bodies are in a straight line, the spine of actual cheetahs is supposed to be at the neutral position when the spine is slightly flexed. In future research, we would like to investigate the effects of such asymmetry properties.

Although galloping is characterized by different foot-contact timings between four legs, our model focused only on different foot-contact timings between the fore and hind legs. Different foot-contact timings between the left and right legs influence the gait stability and performances. In future research, we would like to improve our model to investigate the dynamical effects of different foot-contact timings between four legs on quadrupedal galloping.

Furthermore, although our model does not have any actuator or dissipation, actual cheetahs lose energy through collisions and inject energy via muscles. Moreover, trunk muscles work effectively during acceleration. In future research, we also would like to incorporate these effects to our model to improve the understanding of the mechanism of cheetah galloping.

## Data Availability

The raw data supporting the conclusion of this article will be made available by the authors, without undue reservation.
